# Supplementary material for: Resting-State EEG Alpha Rhythms Are Related to CSF Tau Biomarkers in Prodromal Alzheimer’s Disease
Source: Int J Mol Sci. 2025 Jan 3;26(1):356. doi: 10.3390/ijms26010356 (PMC11720070; doi:10.3390/ijms26010356)
Supplement: Supplementary file 1 [file ijms-26-00356-s001.zip › ijms-3297824-supplementary.pdf]

Resting-State EEG Alpha Rhythms Are Related to CSF Tau Biomarkers in Prodromal Alzheimer's Disease

Supplementary Materials, *Ethical approval statement*

The datasets for the present study were obtained from the international PharmaCog and The PDWAVES Consortium (www.pdwaves.eu) archives. Participants were recruited from various clinical centers, including Sapienza University of Rome (Italy), Institute for Research and Evidence-based Care (IRCCS) "Fatebenefratelli" of Brescia (Italy), IRCCS SDN of Naples (Italy), IRCCS Oasi Maria SS of Troina (Italy), IRCCS Ospedale Policlinico San Martino and DINOGMI (University of Genova, Italy), Hospital San Raffaele of Cassino (Italy), Hospital of Perugia (Italy), Hospital of Chieti (Italy), IRCCS San Raffaele Pisana of Rome (Italy), Medipol University of Istanbul (Turkey), and Dokuz Eylül University of Izmir (Turkey).

Table S1 reports the name of the local institutional ethics committee and the approval code for the units mentioned above.

Table S1. Ethical approval statement. Name of the local institutional ethics committee and approval code for each unit.

CLINICAL UNIT	NAME OF THE LOCAL INSTITUTIONAL ETHICS COMMITTEE AND APPROVAL CODE
Sapienza University of Rome (Italy)	S. Andrea Hospital University Ethical Committee (Rome, Italy), Ref. 73/2012 of May 25, 2017
IRCCS "Fatebenefratelli" of Brescia (Italy)	IRCCS "Fatebenefratelli" Ethical Committee (Brescia, Italy), Prot. n 1024/2011/P of October 10, 2011
IRCCS Synlab SDN of Naples (Italy)	IRCCS Synlab SDN Ethical Committee (Naples, Italy), Prot. 4-12 of September 21, 2012
Oasi Research Institute-IRCCS of Troina (Italy)	IRCCS Sicilia-Oasi Maria SS Ethical Committee (Troina, Italy), Ref. 2022/04/05/CE-IRCCS-OASI/52 of April 5, 2022
IRCCS Ospedale Policlinico San Martino and DINOGMI (University of Genova, Italy)	Liguria Regional Ethics Committee (Genoa, Italy), Ref. 703 of May 31, 2013
Hospital San Raffaele of Cassino (Italy)	Hospital San Raffaele of Cassino Ethical Committee (CE Lazio 2, Italy), Ref. 160-15 of 23 Nov 2016, n.0148158/2016
Hospital of Perugia (Italy)	Regional Umbria Ethical Committee (Perugia, Italy), Prot. n 232 of April 2021

Hospital of Chieti (Italy)	Hospital of Chieti Ethical Committee (Chieti, Italy), Prot. n 2234 of September 2022
IRCCS San Raffaele Pisana of Rome (Italy)	IRCCS San Raffaele Pisana Ethical Committee (Rome, Italy), Ref. 7/2014 of July 29, 2014
Izmir University of Economics, Faculty of Medicine (Turkey)	Dokuz Eylül University Ethical Committee (Izmir, Turkey), Prot no: 3815-GOA, Ref. No. 2018/05-09, of February 15, 2018
Medipol University of Istanbul (Turkey)	Istanbul Medipol University Ethical Committee (Istanbul, Turkey), Ref. 10840098-604.01.01. of May 30, 2018
